# Antigens of *Aeromonas salmonicida* subsp. *salmonicida* specifically induced *in vivo* in *Oncorhynchus mykiss*

**DOI:** 10.1111/jfd.12430

**Published:** 2015-12-11

**Authors:** S Menanteau‐Ledouble, M El‐Matbouli

**Affiliations:** ^1^ Clinical Division of Fish MedicineDepartment for Farm Animals and Veterinary Public HealthUniversity of Veterinary MedicineViennaAustria

**Keywords:** Antigenic profile, AopO, In vivo‐induced antigen technology, lactoylglutathione lyase, LamB, Virulence

Furunculosis is a major fish disease, associated with high mortality rates, and its effect is particularly significant in farmed salmonids (Hiney & Olivier 1999). Efficacious vaccines, generally injected alongside an oil adjuvant, are available. However, certain side effects have been reported (Midtlyng, Reitan & Speilberg 1996; Anderson *et al*. 1997) and protection can lapse in stressed fish and in non‐salmonid species (Gudmundsdóttir & Björnsdóttir 2007).

In vivo‐induced antigen technology (IVIAT) uses antibodies raised by individuals exposed to the pathogen of interest. These antibodies are then adsorbed against an *in vitro* culture of both the pathogen and, to avoid cross‐reaction, the organism in which the genomic library will be expressed (Handfield *et al*. 2000). This removes antibodies binding antigens expressed under culture conditions. The remaining antibodies, recognizing antigens specifically expressed during growth within the host, are used to probe a genomic library expressing random segments from the genome of the pathogen of interest. Reactive clones are sequenced and identified based on homology, identifying genes overexpressed *in vivo* (Rollins *et al*. 2005). Antigens associated with the infectious phenotype are likely to act as virulence factors and constitute interesting targets for vaccine development.

Consequently, we have previously applied IVIAT to *Aeromonas salmonicida* subsp. *salmonicida* (clinical isolate A14390) infecting rainbow trout weighing an average of 92 grams (Menanteau‐Ledouble *et al*. 2014a; Menanteau‐ledouble *et al*. 2014b) in a study approved by the university institutional ethics committee and the national authority according to §26 of the Austrian Law for Animal Experiments (Tierversuchsgesetz 2012–TVG 2012‐91 under the No. GZ 68.205/140‐II/3b/2012). Sera were harvested at multiple time points to sample a wide array of the immune response and screening identified four antigens: UDP‐3‐O‐acyl‐N‐acetylglucosamine deac‐etylase (involved in cell wall synthesis), RNA polymerase sigma factor RpoD (a regulator of gene expression) as well as TonB (that provides energy for transport across the cell membrane) and a hypothetical protein (Menanteau‐ledouble *et al*. 2014b).

In this study, we report on further screening of this library using the same pool of adsorbed sera. A significant difference between both studies is that more time points were included in the RT‐qPCR to confirm that the genes discovered were overexpressed throughout the course of the infection: This time, six time points were included: 1, 6, 12 and 48 h as well as a 1 and 2 weeks post‐infection. Mean fold changes in gene expression were calculated between *in vitro* cultures and the various infected fish tissues according to the 2^−ΔΔC^t method (Livak & Schmittgen 2001).

Four more proteins were detected during this renewed screening and were identified based on sequence homology (Table 1): AopO; lactoylglutathione lyase; a LamB‐like maltoporin; and a hypothetical conserved protein. Each sequence displayed a very high level of homology with genes from the *A. salmonicida* subsp. *salmonicida* A449 genome (Reith *et al*. 2008). These sequences were then further analysed in silico.

**Table 1:** List of the genes identified in this study

Sequence identified	GenBank accession number	Percentage of identity (%)	Query cover (%)
*aopO*	DQ386862	99	93
Lactoylglutathione lyase	CP000644 Region: 1286580 to 1286990	100	94
Hypothetical protein	CP000644 Region: 1072365 to 1073015	99	97
LamB	CP000644 Region: 2545944 to 2547230	99	91

When RT‐qPCRs were performed, they confirmed that all four genes were more highly expressed *in vivo*: the average expression ratio of the four genes was 8.94E^+04^ (±4.69E^+04^) between bacteria in infected tissue samples and bacterial cultures.

The first protein identified was AopO (Genbank identification number: DQ386862). A homologue to the effector protein YopO of *Yersinia ruckeri* (Dacanay *et al*. 2006), AopO is secreted through the type III secretion system (T3SS) (Vanden Bergh *et al*. 2013a Vanden Bergh *et al*. 2013b), a virulence mechanism that is considered particularly important in *A. salmonicida* (Burr *et al*. 2003). Previously, it had been shown that mutants deficient in the expression of three effector proteins of the T3SS (AopO, AopH and AexT) displayed a significantly reduced intracellular survival at 24 HPI in adherent head kidney macrophages (Fast *et al*. 2009). However, inactivation of *aopO* alone only had a moderate effect when the fish were infected by immersion and none during injection challenge (Dacanay *et al*. 2006). Despite being identified in our genomic library, *aopO* is carried on a motile genetic element (Stuber *et al*. 2003). However, the plasmid carrying *aopO* is large, approximately 140 kb, and such large plasmids are difficult to separate from bacterial chromosomes and are often found in genomic preparations.

**Figure 1:**
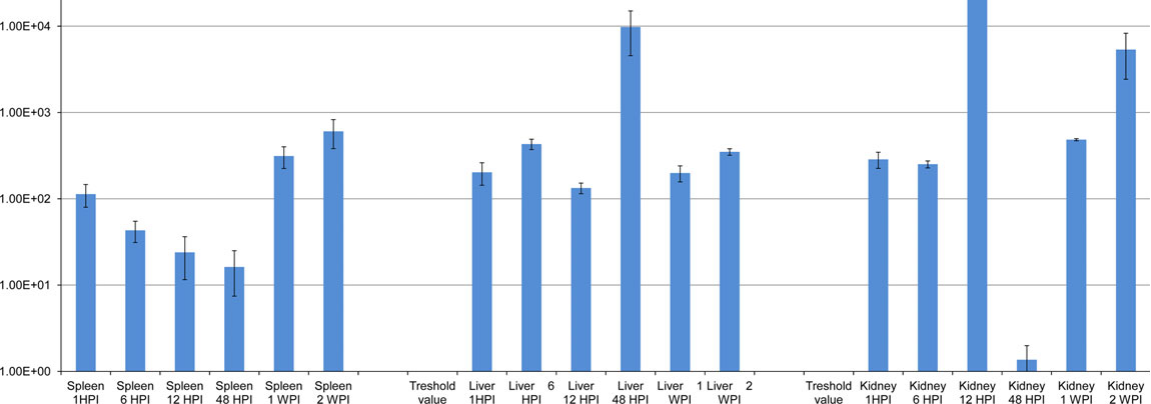
Relative gene expression of *aopO* calculated between the bacteria in the spleens, livers and kidneys of rainbow trout *in vitro*. 16S rRNA expression was used for normalization, and relative gene expression changes were determined. Each value represents the mean of triplicates.

The change in the transcription of *aopO* was 7.04E^+04^ ±3.43E^+04^ in average between infected organs and *in vitro* cultures (Fig. 1), as calculated by the 2^−ΔΔC^t method. Notably, this gene was found not to be significantly overtranscribed in the kidney at 48 HPI (mean fold change of 1.36 ±6.28E^−01^). This was the exception as, otherwise, all investigated genes were found to have significantly higher expression levels in all three organs at every time point compared to the cultures.

**Figure 2:**
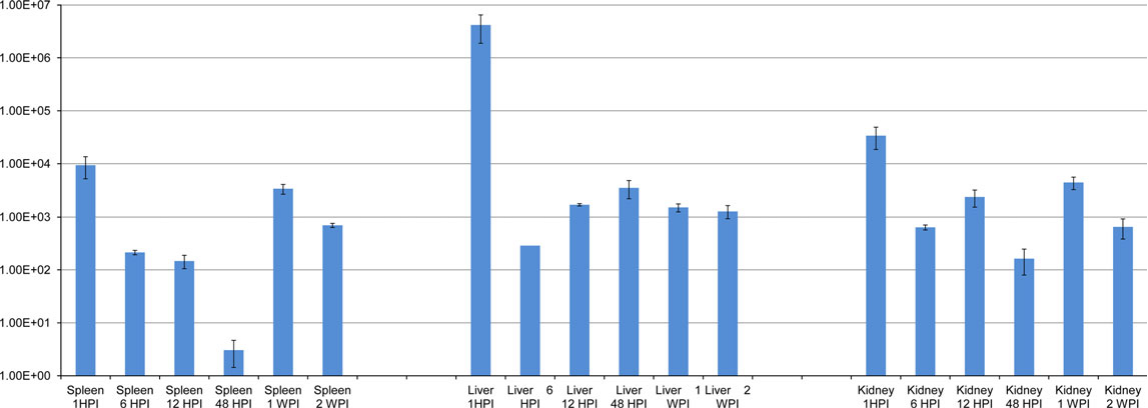
Mean fold change in the expression of the gene coding for lactoylglutathione lyase over the course of this study.

Indeed, the gene coding for lactoylglutathione lyase (CP000644 region: 1286580 to 1286990, belonging to the cluster of orthologous groups (COG) 3324) appeared significantly overexpressed in all three organs sampled and at all time points with an average value of 2.65E^+05^ ±1.74E^+05^ (Fig. 2). Lactoylglutathione lyase is known to be involved in cellular detoxification and resistance to oxidative stress (Alsop & Vijayan 2009), for example in *Lactococcus lactis* (Li *et al*. 2003). In *Streptococcus mutans*, this molecule is overexpressed under acidic condition and is involved in the bacterial resistance to low pH (Korithoski, Lévesque & Cvitkovitch 2007). It has also been shown to play an important role in the intracellular invasiveness of *Salmonella* as well as in the translocation of effectors encoded on the *Salmonella* pathogenicity island 2, and mutants deficient in this enzyme display a reduced invasiveness into epithelial cells (Chakraborty *et al*. 2014).

**Figure 3:**
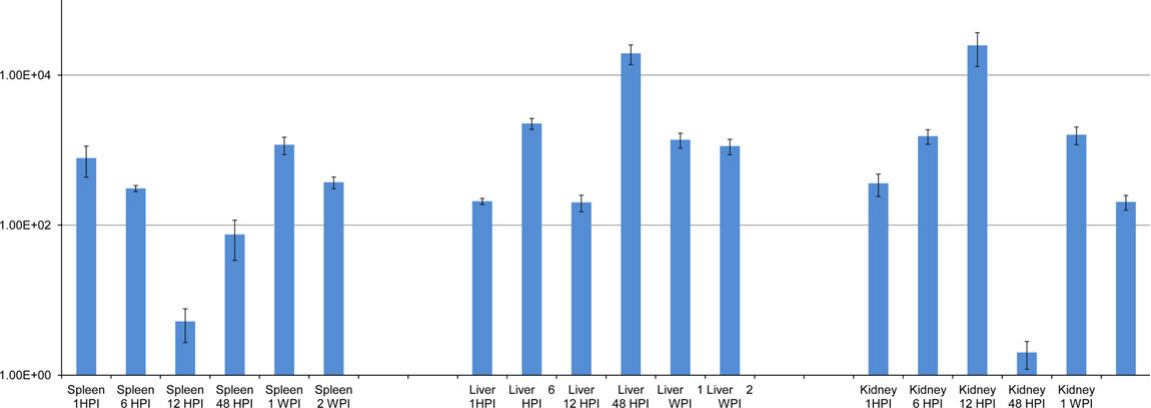
Mean fold change in the expression of the gene coding for the conserved hypothetical protein over the course of this study.

The third protein identified was a conserved hypothetical protein (CP000644 region: 1072365 to 1073015) that has yet to be characterized. The mean fold change in the expression of the gene encoding for this protein was 2.76E^+03^ ±8.16E^+02^ (Fig. 3). PSORTb found that the protein was likely located within the cytoplasm (localization score of 8.96), while Pfam described two endonuclease domains, although their scores were low: 57.2 and 32.1. Indeed, ProtFun found the likelihood of this protein to play an enzymatic role to be low: 0.353.

**Figure 4:**
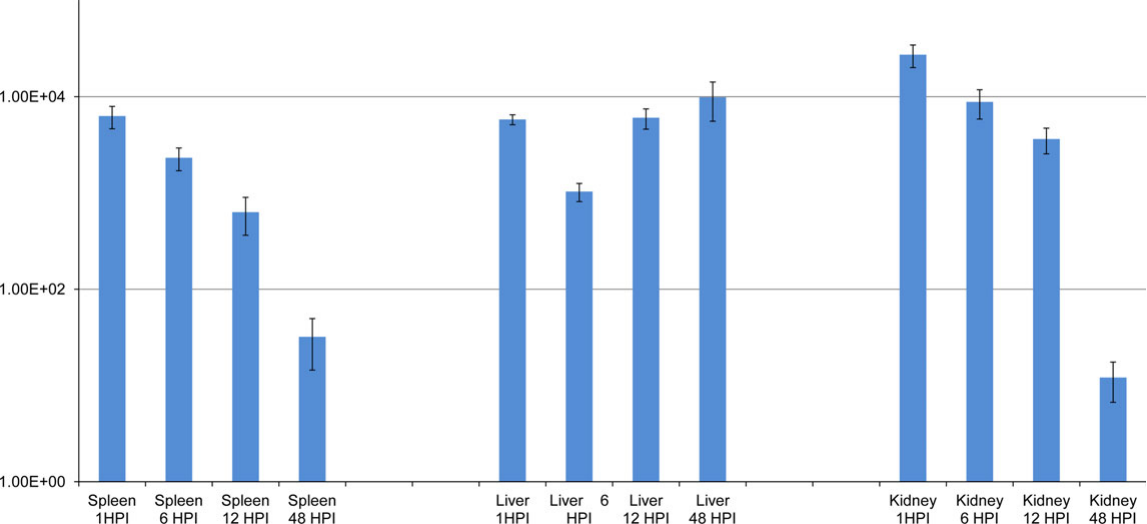
Mean fold change in the expression of *lamB* over the course of this study.

Finally, we identified a LamB‐like maltoporin (CP000644 region: 2545944 to 2547230), belonging to the COG 4580. Unfortunately, because of the low numbers of bacteria at the later time points, only very low copy numbers could be detected for this gene at 1 and 2 weeks post‐infections and the results from the expression ratio analysis appeared as outliers compared to that of the other genes or time points. It was therefore decided to exclude these values from the analysis. After exclusion of these two later time points, the mean fold change of *lamB* was 5.98^+03^ ±1.12E^+03^ (Fig. 4). LamB has been well studied as a specific diffusion channel for the uptake of maltodextrins (Ranquin & Van Gelder 2004). Interestingly, a similar LamB homologue termed Omp48 has been implicated in the binding of *Aeromonas veronii* to the extracellular matrix as well as to HeLa epithelial cells (Vàzquez‐Juárez *et al*. 2004). Moreover, recombinant vaccines that targeted this molecule were found to be protective against infection with *Aeromonas hydrophila* (Khushiramani *et al*. 2012) as well as to offer cross‐protection against *Edwardsiella tarda* and a number of *Vibrio* species (Khushiramani *et al*. 2012; Lun *et al*. 2014).

Two inserts were also sequenced that were identical to the ones previously identified (Menanteau‐Ledouble *et al*. 2014a; Menanteau‐ledouble *et al*. 2014b). The first was identical to the UDP‐3‐O‐acyl‐N‐acetylglucosamine and likely originated from the same original insertion event. The sequence of the other insert (a hypothetical protein) originated from a different segment on the same gene, suggesting that the same gene had been identified independently twice.

Eight genes were identified through both screenings. This relatively low number is expected for IVIAT: this technique only identifies strongly differentially expressed proteins and only if they are sufficiently immunogenic to generate a detectable antibody response.

In the future, it would be interesting to further investigate the significance of these genes in the disease process, for example by inhibiting their expression using defined deletion mutants (Vipond *et al*. 1998) before testing these mutants in an infection challenge. Similarly, the hypothetical protein could be characterized and the vaccine potential of LamB could be investigated.
